# Novel nanowire-structured polypyrrole-cobalt composite as efficient catalyst for oxygen reduction reaction

**DOI:** 10.1038/srep20005

**Published:** 2016-02-10

**Authors:** Xianxia Yuan, Lin Li, Zhong Ma, Xuebin Yu, Xiufang Wen, Zi-Feng Ma, Lei Zhang, David P. Wilkinson, Jiujun Zhang

**Affiliations:** 1 Department of Chemical Engineering, Shanghai Jiao Tong University, Shanghai, 200240, China; 2 Department of Materials Science, Fudan University, Shanghai, 200433, China; 3 The School of Chemistry and Chemical Engineering, South China University of Technology, Guangzhou, 510640, China; 4 Energy, Mining & Environment, National Research Council of Canada, Vancouver, BC V6T 1W5, Canada; 5 Department of Chemical and Biochemical Engineering, University of British Columbia, Vancouver, Canada

## Abstract

A novel nanowire-structured polypyrrole-cobalt composite, PPy-CTAB-Co, is successfully synthesized with a surfactant of cetyltrimethylammounium bromide (CTAB). As an electro-catalyst towards oxygen reduction reaction (ORR) in alkaline media, this PPy-CTAB-Co demonstrates a superior ORR performance when compared to that of granular PPy-Co catalyst and also a much better durability than the commercial 20 wt% Pt/C catalyst. Physiochemical characterization indicates that the enhanced ORR performance of the nanowire PPy-CTAB-Co can be attributed to the high quantity of Co-pyridinic-N groups as ORR active sites and its large specific surface area which allows to expose more active sites for facilitating oxygen reduction reaction. It is expected this PPy-CTAB-Co would be a good candidate for alkaline fuel cell cathode catalyst.

Polymer electrolyte membrane Fuel cells (PEMFCs) are a kind of prospective green power sources that can convert chemical energy in the fuel directly into electricity with high energy efficiency and low/zero emissions^1^,^2^,^3^,^4^. Their performance including energy/power densities and life-time strongly depends on the electrocatalysts for promoting the oxygen reduction reaction (ORR) at cathode. At current state of technology, platinum- and its alloys-based materials are the most efficient and practical ORR catalysts which can give both high electrochemical activity and acceptable durability. However, the scarcity of platinum and its high cost limit fuel cells’ wide and sustainable commercialization.

Currently, there are two major types of PEMFCs, one is the proton exchange membrane fuel cells (also called Acid-PEMFCs) where the membranes are proton conductive, and the other is the Alkaline-PEMFCs where the membranes are hydroxide conductive. In normal, due to the acidic environment of Acid-PEMFCs, their cathode ORR is slower than that in Alkaline-PEMFCs. Thus, Alkaline-PEMFCs may present more opportunities for non-Pt–based electrocatalysts for ORR than that Acid-PEMFCs do. In this sense, Alkaline-PEMFCs are considered to be the feasible and promising alternatives to Acid-PEMFCs in terms of their possible usage of non-noble metal catalysts as well as their better stability of component materials than that in Acid-PEMFCs^5^,^6^,^7^.

Regarding non-noble metal catalysts for PEMFC applications, a wide range of catalyst materials have been explored in the last several decades, including transition metal macrocycles^8^,^9^,^10^, manganese oxides^11^,^12^,^13^, spinel AB_2_O_4_ complexes^14^,^15^,^16^, heat-treated carbon supported transition metal-nitrogen complexes (M-N/C, M = Fe, Co, Mn, etc.)^17^,^18^,^19^, perovskite-type oxides^20^,^21^,^22^ and metal free catalysts^23^,^24^,^25^. Among them, the M-N/C catalysts have been paid great attentions owing to their higher activities and four-electron-transfer selectivity towards ORR. As a representative member of M-N/C catalysts, polypyrrole (PPy)-based cobalt-contained catalyst has been investigated in recent years as ORR catalyst in alkaline media and demonstrated promising performance^26^,^27^. Even the metal free pristine PPy and pyrolyzed PPy have also been investigated as ORR catalysts in acidic solutions^28^,^29^. Depending on the preparation conditions, PPy could be formed into various morphologies^30^, such as film^31^, nanoparticle^32^, nanotube array^33^, nanowire^34^,^35^, microtube^36^, hollow structure^37^ and so on. The morphology of PPy has been identified to influence its ORR performance as the metal free catalyst. Morozan *et al*.^38^ reported that pyrolyzed PPy with tubules-like morphology could exhibit a much better ORR catalytic performance in 0.1 M KOH than that with granular-like morphology. However, the morphology effects of metal-contained PPy catalysts on their ORR performance in either alkaline or acidic media have seldom been seen in literature.

In this work, a nanowire-structured PPy-Cobalt composite was synthesized using nanowire polypyrrole and cobalt acetate by surface immobilization of cobalt ions followed by a pyrolysis in an inert atmosphere, its ORR performance in alkaline solution was electrochemically evaluated and compared with that of the PPy-Co granules. Experiment results showed that this novel catalyst had a superior ORR performance compared to a granular PPy-Co catalyst and also a better durability than commercial 20% Pt/C catalyst. To understand the enhancement effect of this catalyst for ORR, some physiochemical characterizations, including X-ray diffraction (XRD), scanning electron microscopy (SEM), transmission electron microscopy (TEM), X-ray photoelectron spectroscopy (XPS), N_2_ adsorption-desorption isotherm and Inductive Coupled Plasma Emission Spectrometer (ICP), were employed. It is concluded that the ORR enhancement effect of such a catalyst is due to the resulted Co-pyridinic-N active sites for ORR and also a larger specific surface area with more active site exposure.

## Results

### Synthesis and characterization of catalysts

**Figure 1:**
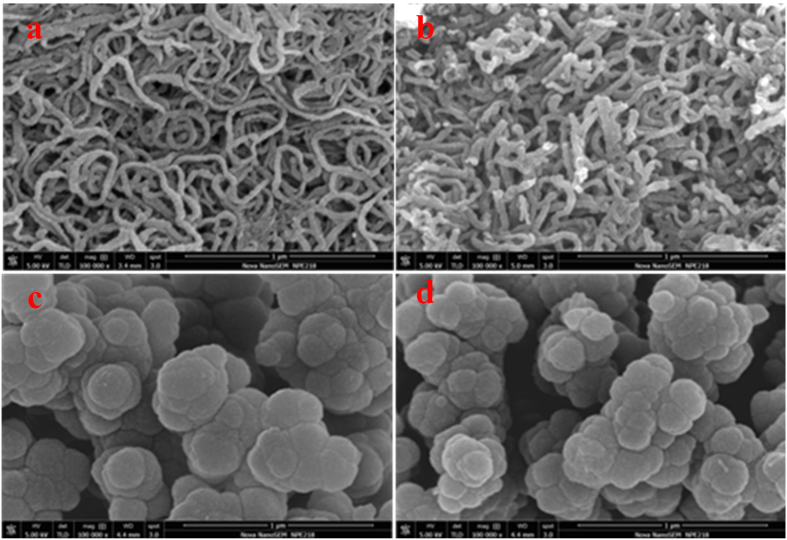
SEM images of PPy-CTAB (**a**), PPy-CTAB-Co (**b**), PPy (**c**), and PPy-Co (**d**).

**Figure 2:**
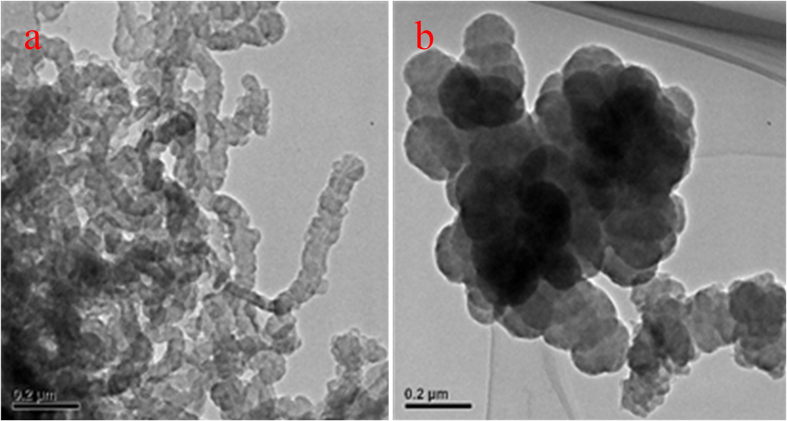
TEM images of PPy-CTAB-Co (**a**) and PPy-Co (**b**) catalysts.

SEM images were taken to observe morphologies of the as-prepared polypyrrole and the cobalt-anchored catalysts. Figure 1a identifies nanowire morphology of the PPy-CTAB prepared with CTAB as the surfactant, its nanowire structure can still be maintained after the addition of Co metal and the pyrolysis process, as shown in Fig. 1b for the PPy-CTAB-Co catalyst. However, the average nanowire size was slightly increased probably owing to the agglomeration of nanowire PPy during the pyrolysis. In contrast, a granular morphology can be observed for both PPy (Fig. 1c) which was prepared without surfactant and the cobalt-contained catalyst of PPy-Co (Fig. 1d), but the average size of the PPy-Co catalyst is slightly smaller, which could be ascribed to the decomposition of PPy and the structure re-configuration during the pyrolysis. Both the nanowire and granular structures of PPy-CTAB-Co and PPy-Co catalysts could also be observed with the TEM images (Fig. 2).

### Electrochemical performance of the catalysts towards ORR

**Figure 3:**
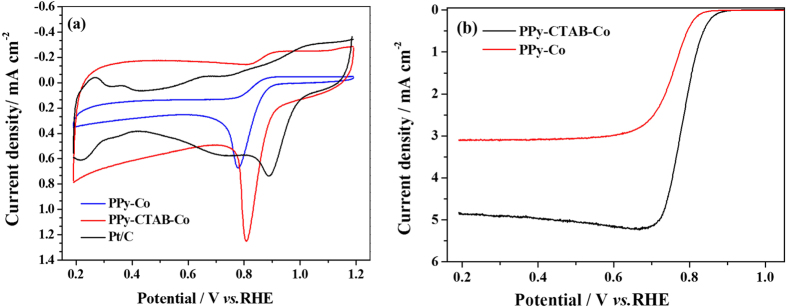
Cyclic voltammograms (CVs) (**a**), and I-V polarization curves at 1600 rpm (**b**) of PPy-CTAB-Co and PPy-Co catalyzed glassy carbon electrodes in O_2_-saturated 0.1 M KOH solution. Potential scanning rates for both CVs and I-V curves are 5 mV s^−1^; Catalyst loadings: 0.40 mg cm^−2.^

The catalytic ORR activity of the nanowire-structured PPy-CTAB-Co was evaluated in O_2_-saturated 0.1 M KOH electrolyte with CV in a three-electrode cell at 5 mV s^−1^, the obtained result is shown in Fig. 3a, where the CV curves of both PPy-Co granules and 20 wt% Pt/C catalysts are also shown for comparison. Although the ORR activities of both nanowire PPy-CTAB-Co and granular PPy-Co catalysts are apparently lower than that of 20 wt% Pt/C catalyst as seen from the ORR peak potentials, the activity of PPy-CTAB-Co is, however, significantly higher than that of PPy-Co. Its ORR peak at about −0.182 V is apparently more positive than that of −0.213 V for the granular PPy-Co. Moreover, the ORR peak current density of PPy-CTAB-Co is about twice higher than that for PPy-Co. These results imply the higher ORR catalytic activity of nanowire PPy-CTAB-Co than granular PPy-Co catalyst. Figure 3b presents the polarization curves of the PPy-CTAB-Co and PPy-Co catalysts measured at an electrode rotating rate of 1600 rpm with a potential scanning rate of 5 mV s^−1^. Both the onset and half wave potentials of PPy-CTAB-Co catalyst are obviously higher than that of granular PPy-Co, indicating again the higher catalytic activity of the nanowire PPy-CTAB-Co.

**Figure 4:**
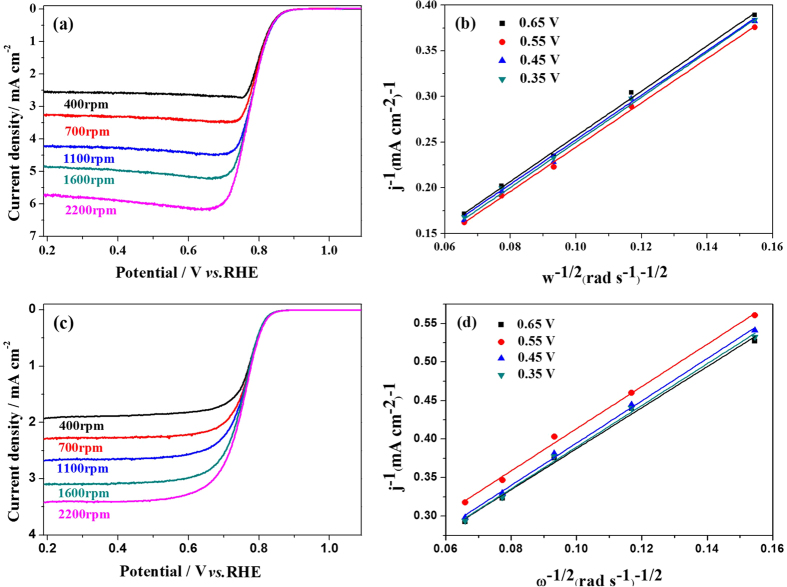
RDE I-V polarization curves recorded on glassy carbon electrodes coated by PPy-CTAB-Co (**a**) and PPy-Co (**c**) in O_2_-saturated 0.1 M KOH solution, respectively. Potential scannig rate is 5 mV s^−1^; Catalyst loadings: 0.40 mg cm^−2^. K-L plots (**b,d**) of data in (**a,b**).

To quantitatively investigate the ORR performance of the nanowire PPy-CTAB-Co catalyst, Fig. 4a gives the RDE curves measured at various electrode rotating rates along with that of PPy-Co shown in Fig. 4c for comparison. Generally, the number of transferred electrons (*n*), the so-called 4-electron pathway selectivity, for ORR can be calculated with Koutecky-Levich (K-L) equation^39^:


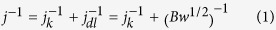



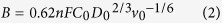


where *j* is the disk current density, *j*_k_ is the kinetic current density, *j*_dl_ is the diffusion current density, *F* is the Faraday constant, *C*_0_ is the concentration of O_2_ in the electrolyte, *D*_0_ is the diffusion coefficient of O_2_ in the electrolyte, *v*_0_ is the kinetic viscosity of the electrolyte, and *w* is the rotating rate of the disk electrode. The K-L plots derived from the current densities at 0.65, 0.55, 0.45 and 0.35 V (*vs*. RHE) are shown in Fig. 4b,d giving the electron-transfer numbers of 3.71 and 3.35 for PPy-CTAB-Co and PPy-Co, respectively. This indicates that both the nanowire PPy-CTAB-Co and granular PPy-Co undergo 4-electron-transfer dominated ORR, but the former has higher 4-electron pathway selectivity.

### Stability of the catalysts

**Figure 5:**
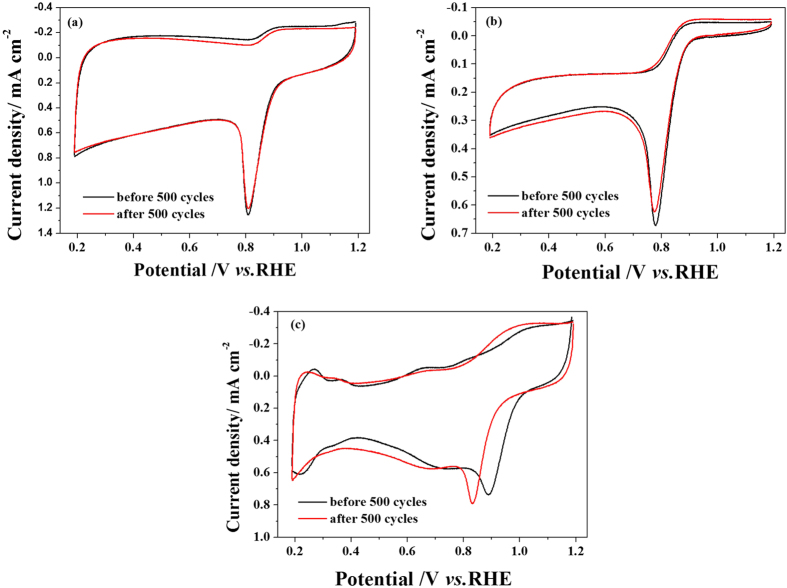
Stability of electrodes coated by PPy-CTAB-Co (**a**), PPy-Co (**b**) and 20wt% Pt/C (**c**), respectively, measured in O_2_-saturated 0.1 M KOH solution. Potential scannig rate is 5 mV s^−1^; Catalyst loadings: 0.40 mg cm^−2.^

Stability is a necessary property for fuel cell catalysts. In the present work, the stability of nanowire PPy-CTAB-Co and granular PPy-Co catalysts were evaluated by comparing the CV curves in O_2_-saturated 0.1 M KOH before and after 500 CV cycles in Ar-saturated 0.1 M KOH, and that of the 20 wt% Pt/C was also measured for comparison, the results are shown in Fig. 5. Excellent stability can be observed for the nanowire PPy-CTAB-Co catalyst, almost no decay could be found in its ORR peak potential, while the granular PPy-Co catalyst shows a slight decrease in ORR activity. For the commercial 20wt%Pt/C catalyst, however, its ORR peak potential is drastically decreased by 60 mV after 500 cycles. These results indicate the better stability of both PPy-CTAB-Co and PPy-Co catalysts than the 20 wt% Pt/C catalyst and the nanowire PPy-CTAB-Co catalyst can give the best stability.

## Discussions

**Figure 6:**
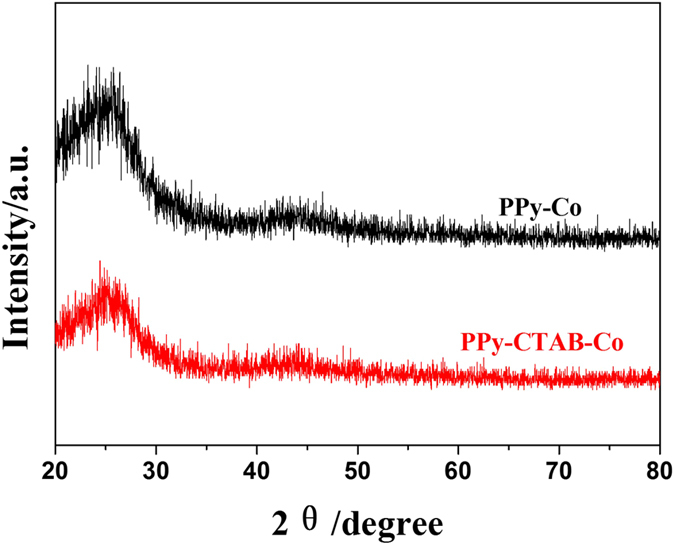
XRD patterns of PPy-CTAB-Co and PPy-Co catalysts.

In order to understand the superior ORR performance of the nanowire PPy-CTAB-Co catalyst, diverse physiochemical characterizations were employed. Closely similar XRD patterns for PPy-CTAB-Co and PPy-Co catalysts were obtained (Fig. 6) with two broad diffraction peaks at 2θ of about 25° and 43°, which correspond to (002) and (101) phase of carbon, respectively, but none characteristic peaks for metallic cobalt or cobalt oxide could be observed in both catalysts, agreeing well with the SEM/TEM results (Figs 1 and 2) of none Co/CoO particles, probably suggesting that the Co species are not in a crystalline state. Actually the ICP measurements could detect a cobalt content of 4.356 wt% and 0.676 wt% in the catalysts of PPy-CTAB-Co and PPy-Co, respectively. XPS experiments (Fig. S1, Supporting Information) could also display surface cobalt content of 1.03 at% (PPy-CTAB-Co) and 0.24 at% (PPy-Co) in the catalysts, respectively. Consulting with our previous research^40^, these results imply that the cobalt in the catalysts are bonded to nitrogen as Co-N structure, but not exist as metallic cobalt or its oxide.

Fig. S2 in Supporting Information displays the N_2_ adsorption-desorption isotherm of PPy-CTAB, PPy and the resulted PPy-CTAB-Co and PPy-Co catalysts. The calculated BET surface areas for PPy-CTAB and PPy are 120 and 12 m^2^ g^−1^, respectively. This may explain why PPy-CTAB-Co catalyst has a higher cobalt content, as measured with both ICP and XPS discussed above. It is believed that the higher specific surface area of nanowire PPy-CTAB should be able to help absorbing more cobalt ions during cobalt acetate impregnation to form Co-N structure in the final catalyst^41^.

**Figure 7:**
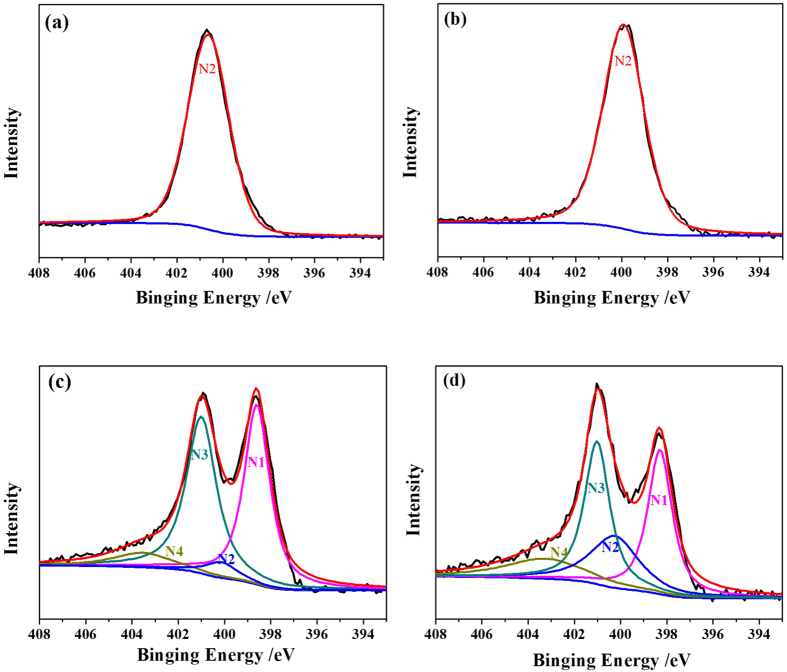
N 1 s XPS spectra for PPy-CTAB (**a**), PPy (**b**), PPy-CTAB-Co (**c**), and PPy-Co (**d**).

N 1 s core level XPS spectra in PPy-CTAB, PPy, and the PPy-CTAB-Co and PPy-Co catalysts were captured with XPS and demonstrated in Fig. 7. In both nanowire PPy-CTAB and granular PPy, the nitrogen is dominated by a main peak of pyrrolic-N (N2) in the range from 400.1 to 400.9 eV, which can be assigned to nitrogen atoms in PPy ring. In both the catalysts of PPy-CTAB-Co and PPy-Co, however, the form of nitrogen has changed drastically because of the decomposition of PPy and structure re-configuration during high temperature pyrolysis. Actually, the nitrogen in both of the catalysts could be deconvoluted into pyridinic-N (N1, 398.0–399.5 eV), pyrrolic-N (N2, 400.1–400.9 eV), quaternary-N (N3, 401–402 eV) and oxidative-N (N4, 402–410 eV)^40^, as shown in Fig. 7. According to Higgins *et al*.^42^, pyridinic-N could provide an edge plane exposure to readily facilitate the adsorption of oxygen, leading to improved ORR performance. Sha *et al*.^40^,^43^ also discussed that pyridinic nitrogen could play a significant role in the ORR performance of pyrolyzed carbon supported polypyrrole-cobalt catalysts with the active sites of Co-pyridinic-N group. In this work, the evaluated concentrations of various types of nitrogen in the studied PPy-CTAB-Co and PPy-Co catalysts are listed in Table 1. It can be seen that the concentration of pyridinic-N in PPy-CTAB-Co is obviously higher than that in PPy-Co. Combined with the higher cobalt content in PPy-CTAB-Co as discussed above and the larger nitrogen content (11.38 at% compared to 8.46 at% for PPy-Co) acquired with XPS spectra (Fig. S1, Supporting Information), it is inferred that the more Co-pyridinic-N structure as ORR active sites in the nanowire PPy-CTAB-Co catalyst is a main factor responsible for the enhanced ORR performance. Moreover, the BET surface areas of PPy-CTAB-Co and PPy-Co catalysts can be calculated using the data in Fig. S2 (Supporting Information), which are 72.2 and 24.4 m^2^ g^−1^, respectively. It is another possible reason for the higher catalytic ORR activity of PPy-CTAB-Co than PPy-Co, because more Co-pyridinic-N active sites exist on the larger surface of PPy-CTAB-Co catalyst to facilitate the oxygen reduction reaction.

## Conclusions

In summary, PPy-CTAB-Co catalyst with a nanowire morphology is successfully synthesized with CTAB as a surfactant and it is tested for catalyzing ORR in alkaline solution. Both much better catalytic ORR activity and higher four-electron-transfer selectivity as well as higher stability than the granular PPy-Co catalyst are observed. Although this catalyst’s ORR activity is slightly inferior to that of the commercial 20 wt% Pt/C catalyst, a much better stability than the Pt/C catalyst in alkaline solution is achieved. With the help of physicochemical techniques, the enhanced ORR performance of nanowire PPy-CTAB-Co can be attributed to large quantity of Co-pyridinic-N groups as ORR active sites and its larger specific surface area which allows more active sites to expose for facilitating oxygen reduction reaction.

## Methods

### Synthesis of catalysts

In order to prepare nanowire PPy, cetyltrimethylammounium bromide (CTAB) was used as the surfactant. The typical procedure^44^ is as follows: 1.5 mmol CTAB was stirred in 150 ml deionized water at 40 ^o^C to form a homogeneous solution, then this solution was cooled down to 0–3 ^o^C to form a CTAB crystalline suspension followed by the addition of pre-cooled ammonium peroxydisulfate (APS) aqueous solution (4.5 mmol APS dissolved in 25 ml deionized water). After 1 minute, 500 μl freshly distilled pyrrole was injected and the obtained mixture was stirred for another 24 hours at 0–3 ^o^C. The resulted mixture was washed with water and ethanol alternately for several times, and then dried under vacuum at room temperature to obtain the PPy-CTAB with a nanowire morphology.

For comparison, granular PPy with small particles was also prepared: 2 ml freshly distilled pyrrole was dissolved in 100 ml deionized water, followed by an addition of APS aqueous solution (57.6 mmol APS dissolved in 200 ml deionized water). After a stirring for 24 hours, the mixture was washed several times with water and ethanol alternately, and then dried under vacuum at room temperature to obtain the granular PPy.

In two individual experiments, 0.1 g PPy-CTAB or PPy was added into 300 ml saturated cobalt acetate solution, separately. The obtained mixture was stirred and refluxed at 60 ^o^C for 10 hours, and then washed with excessive deionized water and dried under vacuum at 60 ^o^C. The resulted black powders were then pyrolyzed in argon atmosphere at 800 ^o^C for 2 hours to obtain the final catalysts of PPy-CTAB-Co and PPy-Co, respectively.

Commercial 20 wt% Pt/C catalyst from BASF company was used as the baseline for ORR performance comparison.

### Characterizations

Powder XRD patterns were obtained on a Shimadzu 6000 X-ray diffract meter using Cu *K*α radiation (*λ* = 1.5406 Å). SEM images were recorded on a NOVA NANOSEM 450 instrument. TEM images were recorded on a JEOL JEM-2100 instrument operating at 200 kV and 30 mA. XPS measurements were performed on Kratos AXIS ULTRA DLD with Al *K*α as excitation source (*hv* = 14 kV). The N_2_ adsorption-desorption isotherms were measured using Micromeritics ASAP 2010 M + C instrument at 77 K to calculate the Brunauer-Emmett-Telley (BET) specific surface areas. Cobalt content in the catalysts was detected using a Thermal iCAP 6000 ICP spectrometer by soaking the catalysts in aqua regia.

### Electrochemical measurements

The electrochemical activities of the catalysts towards ORR were evaluated in a three-electrode cell controlled by a CHI 750 A potentiostat/galvanostat. A platinum wire and a saturated calomel electrode (SCE) were used as the counter electrode and reference electrode, respectively. A rotating disk electrode employing a glassy carbon disk (∅ = 4 mm) was used as the working electrode. 0.1 M KOH was the electrolyte.

The catalyst layer on the working electrode was prepared by pipetting 10 μl catalyst ink onto the glassy carbon disk and dried at room temperature. For catalyst ink preparation, 5.0 mg catalyst sample was ultrasonically dispersed in a solution containing 50 μl Nafion^®^ solution (5 wt%, DuPont) and 950 μl deionized water.

Cyclic voltammograms (CVs) of the catalysts were recorded between 0.19 and 1.19 V (*vs.* RHE) in O_2_-saturated 0.1 M KOH at 25 °C with a potential scanning rate of 5 mV s^−1^. The rotating disk electrode (RDE) experiments were carried out in an alkali O_2_- and Ar-saturated solution (0.1 M KOH), respectively, with the same conditions as CV at various electrode rotating rates.

## Additional Information

**How to cite this article**: Yuan, X. *et al*. Novel nanowire-structured polypyrrole-cobalt composite as efficient catalyst for oxygen reduction reaction. *Sci. Rep.* **6**, 20005; doi: 10.1038/srep20005 (2016).

## Supplementary Material

Supplementary Information

## Figures and Tables

**Table 1:** Concentrations of various nitrogen groups in the catalysts of PPy-CTAB-Co and PPy-Co.

	PPy-CTAB-Co	PPy-Co
Pyridinic-N (N1)	0.4333	0.3606
Pyrollic-N (N2)	0.0464	0.0892
Quaterary-N (N3)	0.4415	0.4305
Oxidative-N (N4)	0.0788	0.1197
