# Supplementary material for: Novel nanowire-structured polypyrrole-cobalt composite as efficient catalyst for oxygen reduction reaction
Source: Sci Rep. 2016 Feb 10;6:20005. doi: 10.1038/srep20005 (PMC4748222; doi:10.1038/srep20005)
Supplement: Supplementary Information [file srep20005-s1.pdf]

Supporting Information for

Novel nanowire-structured polypyrrole-cobalt composite as efficient catalyst for oxygen reduction reaction

Xianxia Yuan*[,a], Lin Li[a], Zhong Ma[a], Xuebin Yu[b], Xiufang Wen[c], Zi-Feng Ma[a], Lei Zhang[d], David P. Wilkinson[e] and Jiujun Zhang[d]

[a] Department of Chemical Engineering, Shanghai Jiao Tong University, Shanghai, 200240, China

[b] Department of Materials Science, Fudan University, Shanghai, 200433, China

[c] The School of Chemistry and Chemical Engineering, South China University of Technology, Guangzhou, 510640, China

[d] Energy, Mining & Environment, National Research Council of Canada, Vancouver, BC V6T 1W5, Canada

[e] Department of Chemical and Biochemical Engineering, University of British Columbia, Vancouver, Canada

*** Corresponding author:** yuanxx@sjtu.edu.cn (X. Yuan)

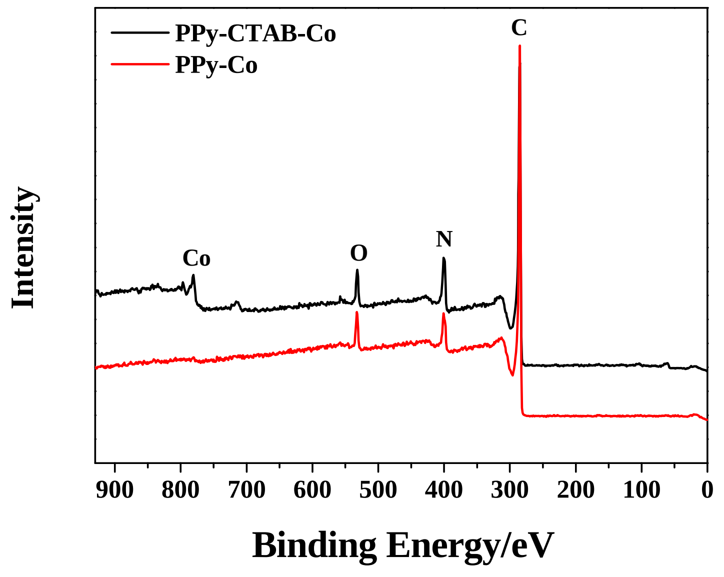

Fig. S1 XPS spectra of the PPy-CTAB-Co and PPy-Co catalysts

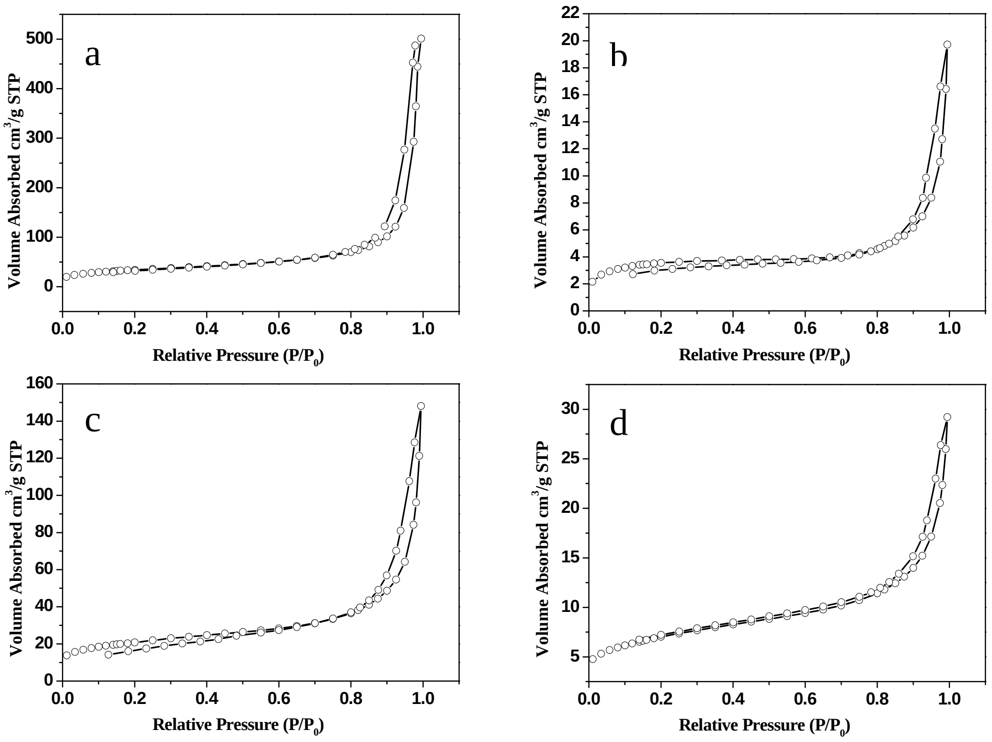

Fig. S2 N_2 adsorption-desorption isotherm of PPy-CTAB (a), PPy (b), PPy-CTAB-Co (c) and PPy-Co (d)
